# Insulin-like growth factors in embryonic and fetal growth and skeletal development (Review)

**DOI:** 10.3892/mmr.2014.2258

**Published:** 2014-05-21

**Authors:** GEORGIOS D. AGROGIANNIS, STAVROS SIFAKIS, EFSTRATIOS S. PATSOURIS, ANASTASIA E. KONSTANTINIDOU

**Affiliations:** 1 1st Department of Pathology, School of Medicine, University of Athens, Athens, Greece; 2 Department of Obstetrics and Gynecology, University Hospital of Heraklion, Crete, Greece

**Keywords:** insulin-like growth factors, insulin-like growth factor-I, insulin-like growth factor-II, fetal growth, skeletal development

## Abstract

The insulin-like growth factors (IGF)-I and -II have a predominant role in fetal growth and development. IGFs are involved in the proliferation, differentiation and apoptosis of fetal cells *in vitro* and the IGF serum concentration has been shown to be closely correlated with fetal growth and length. IGF transcripts and peptides have been detected in almost every fetal tissue from as early in development as pre-implantation to the final maturation stage. Furthermore, IGFs have been demonstrated to be involved in limb morphogenesis. However, although ablation of *Igf* genes in mice resulted in growth retardation and delay in skeletal maturation, no impact on outgrowth and patterning of embryonic limbs was observed. Additionally, various molecular defects in the *Igf1* and *Igf1r* genes in humans have been associated with severe intrauterine growth retardation and impaired skeletal maturation, but not with truncated limbs or severe skeletal dysplasia. The conflicting data between *in vitro* and *in vivo* observations with regard to bone morphogenesis suggests that IGFs may not be the sole trophic factors involved in fetal skeletal growth and that redundant mechanisms may exist in chondro- and osteogenesis. Further investigation is required in order to elucidate the functions of IGFs in skeletal development.

## 1. Introduction

The components of the insulin-like growth factor (IGF) system, include IGFs (IGF-I and IGF-II), type 1 and type 2 IGF receptors, a family of six secreted IGF-binding proteins (IGFBPs) and IGFBP proteases (1). The IGFs are single-chain mitogenic polypeptides, structurally similar to proinsulin, that function in an autocrine/paracrine manner and also as classical hormones. The two IGF receptors are structurally and functionally unrelated. IGF ligand signaling is mediated by IGF-1R, which is a transmembrane glycoprotein with tyrosine kinase activity (2). IGF-2R is a single-chain protein without kinase activity (3). IGF-1R binds IGF-I with up to 20-fold higher affinity than for IGF-II, while IGF-2R strongly binds IGF-II, but barely recognizes IGF-I (2,3). In biological fluids, IGFs are usually bound by members of the secreted IGFBP family, of which the exact role remains unknown. IGFBPs may serve as transport serum proteins, as IGF presentation molecules to the IGF receptors, as molecules prolonging IGF half-life or as a means for tissue-specific IGF localization. In addition, IGFBPs are considered to mediate IGF-independent actions via their own receptors (1). The *Igf2* and *Igf2r* genes are imprinted, expressed in a monoallelic manner depending on parental legacy. In the murine embryo, only the paternal *Igf2* allele is expressed, while only the maternal *Igf2r* allele is expressed (4). However, subsequent to birth, *Igf2* expression becomes biallelic in certain tissues, for example, in the liver (5). The present review focuses on the role of IGF-I in fetal growth and development, paying particular attention to skeletal development.

## 2. Role of IGFs in fetal growth

In initial IGF studies, the predominant roles of IGF-I and -II in fetal growth were elucidated by abundant but largely indirect evidence. IGFs were shown to act as proliferation and differentiation factors in cultured fetal cells (6–8) and preimplantation embryos (9), and were demonstrated to be secreted by cultured fetal cells and explants *in vitro* (10–11).

Direct evidence of the importance of IGFs and IGF receptors in the regulation of embryonic and early postnatal growth was provided by a series of studies using gene knockout, analyzing the phenotypes manifested by mutations, alone or in combination (4). *Igf2*(−/−) nullizygotes and heterozygous mice carrying a paternally derived mutated *Igf2* gene were phenotypically indistinguishable (12). The mice were viable dwarfs with a birth weight 60% that of normal. Ablation of the *Igf1* gene (*Igf1* nullizygotes) resulted in a similar reduction of fetal growth (13) contradicting the prevailing hypothesis that IGF-II was the predominant mediator of fetal growth. Furthermore, the growth deficiency of the *Igf1* mutants became evident at mouse embryonic day E13.5, when the size of the mutant embryos was ~90% that of normal size, subsequent to which the *Igf1*(−/−) embryos continued to grow at a slower rate, thus the mice were ~60% of normal size at the end of gestation (14). Surviving mutants continued to grow postnatally at a retarded rate, resulting in gaining only 30% of normal body weight as adults (14). This is in contrast to the normal birth weights observed in mice with GH-deficiency or GH-resistance (15,16) and suggests that in prenatal mice, IGF-I is secreted independently of GH. Nevertheless, evidence indicates that GH acts as a local growth, differentiation and cell survival factor in the embryo, independent of IGF-I (17). *Igf1r* nullizygotes exhibited an even greater reduction in birthweight (45% of normal) and died immediately following birth (13). The proposed underlying mechanism for growth retardation of *Igf1* knock-out mice is that IGF-I and -II are not mitogenic *per se*. Deletion of *Igf1* is suggested to lead to elongation of cell cycle time, resulting in fewer proliferation events during the same period and the generation of fewer cells than those required for the completion of embryonic development. In addition, evidence provided by Walenkamp *et al* (18) and Bhakta *et al* (19) show that the influence of IGF-1 on fetal growth is dose-related.

## 3. Expression levels of IGF genes and proteins in fetal serum and tissues

The two IGFs have been detected in the fetal plasma early in gestation in the majority of animal species investigated thus far (20–22), with plasma concentrations of IGF-II found to be several fold higher than those of IGF-I (20,22). Notably, high IGF-II concentrations in fetal serum were demonstrated to decline within days following birth (20,23), while serum concentrations of IGF-I appeared to be low in the fetus and rise in the immediate postnatal period, primarily as a result of the onset of GH-stimulated IGF-I production by the liver (20,24).

In accordance with the findings regarding plasma concentrations of IGF-II, the majority of studies reported higher abundance of *Igf2* mRNA in fetal tissues compared with adult tissues (25). This raised the suggestion that IGF-II is the IGF that mediates growth and differentiation in developing fetal tissues. However, while IGF-II was revealed to be more abundant than IGF-I within the conceptus (serum and tissues), IGF-I was most closely associated with fetal growth in the majority of species. Thus, the plasma concentration of IGF-I, but not IGF-II, was found to correlate positively with fetal size and length, as well as birth and placental weight in humans (26–29). Alterations in the plasma or serum concentrations of IGF-I and IGFBP-1 and -3 have been identified in pregnancies complicated by preeclampsia and intrauterine growth restriction, where placental function is inadequate and fetal growth reduced (30–33). In such complicated pregnancies, the placental expression levels of IGF-I and IGFBP-1 are also decreased (34,35).

Since serum concentrations may not reflect the production of peptides in specific tissues, several studies have attempted to detect the expression levels of *Igf* genes and/or peptides *in vivo*. Using reverse transcription-polymerase chain reaction (RT-PCR), transcripts of *Igf* and *Igf* receptor genes were detected in the fetal tissues of various species between the earliest stage of pre-implantation and the final phase of tissue maturation (36–39), while sensitive hybridization methods have shown that *Igf* gene expression was present in almost all human and rodent fetal tissues (40), including the liver, pancreas and osteochondrous tissue.

**Figure 1:**
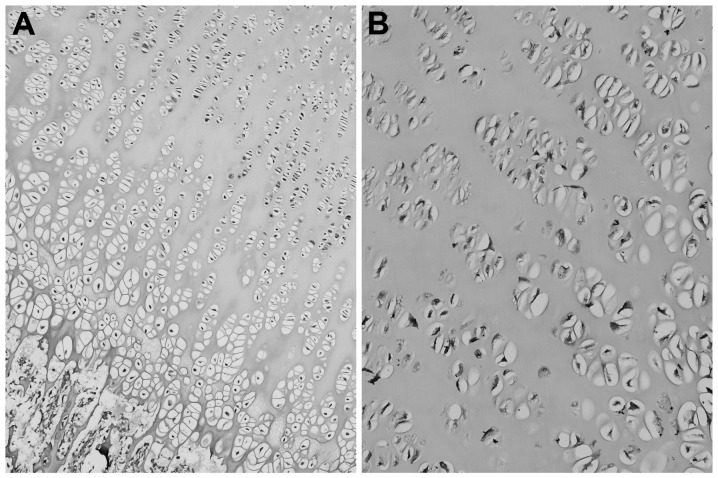
Human femur of a 22-week-gestation fetus: Insulin-like growth factor (IGF)1 cytoplasmic expression is localized in the chondrocytes of all layers in the growth plate. IGF1 immunohistochemical stain. (A) Magnification, ×40. (B) Magnification, ×200.

Previous studies regarding the distribution of IGFs in the bones of piglets and mice, revealed localization within the growth plate (41,42). *Igf1* and *Igf2* mRNA was expressed throughout all zones, albeit *Igf1* less extensively. Immunohistochemical techniques also revealed the expression of IGFs within the resting zone, the hypertrophic zone and the proliferative zone of the growth plate (41) (Fig. 1). Additionally, with the use of RT-PCR, IGFs were also detected within the perichondrium and metaphyseal bone in rats (43).

## 4. IGF-I in limb morphogenesis

During mammalian embryogenesis, growth factors are important not only in cellular proliferation and differentiation but also in morphogenesis. The developing limb constitutes an attractive model of tissue morphogenesis. At the end of week 4 of gestation, the developing limb buds become visible as outpocketings from the ventrolateral body wall. Initially, the limb buds consist of a mesenchymal core derived from the lateral plate mesoderm that forms the bones and connective tissues of the limb, covered by a layer of ectoderm. The ectoderm at the distal border of the limb thickens and forms the apical ectodermal ridge (AER) (44). This ridge exerts an inductive influence on the adjacent mesenchyme, causing the mesenchyme to remain as a population of undifferentiated, rapidly proliferating cells, the progressing zone. As the limbs grow, cells farther from the influence of the AER begin to differentiate into cartilage and muscle. In this manner, development of limb proceeds proximodistally (44). Fingers and toes are formed when cell death in the AER separates the ridge into five parts. The zone of polarizing activity (ZPA) is an additional signaling region at the posterior margin of the limb mesenchyme that controls the antero-posterior patterning of the limb (45).

While the external shape is being established, the mesenchyme in the buds begins to condense and differentiate into chondrocytes. By week 6 of development, the first hyaline cartilage models, foreshadowing the bones of the extremities, are formed by these chondrocytes. Ossification of the bones of the extremities, endochondral ossification, begins by the end of the embryonic period. Primary ossification centers are present in all long bones of the limb by week 12 of development (44).

Several studies have demonstrated the predominant role of the IGFs in limb development. IGF-I has been demonstrated to stimulate proliferation of dissociated limb mesenchymal cells (46), isolated human fetal chondocytes (6), and explanted limb buds of rat and chicken embryos *in vitro* (47,48). Other studies, using *in situ* hybridization and immunohistochemistry, have demonstrated that IGF-I and its receptor (IGF-1R) are expressed *in vivo* by the sub ridge mesodermal cells of the developing rat and chicken limb buds (49–52), while in mouse embryos IGF-I has also been detected in the progress zone (53), suggesting that IGF-I may be involved in promoting the proliferation and outgrowth of the limb mesoderm in response to the AER or ZPA regions. Furthermore, several studies have revealed *Igf1* transcripts in the condensing central core of mouse and chicken limbs (52,53), which implicates IGF-I in the regulation of chondrogenic differentiation. However, other studies did not detect *Igf1* transcripts, and reported only *Igf2* and *Igf1r* transcripts in the undifferentiated mesenchymal condensations and differentiated chondrocyte precursors in murine fetus chondrogenesis (54), verifying the results of *in vitro* experiments in limb organ cultures (47,48,52). *Igf1* transcripts have been reported to be present in the osteoblast, osteo- and chondoclasts and nascent matrix of the long bones of developing chicken and mouse limbs, a location consistent with a potential role for IGF-I in endochondral bone formation (49). Notably, during the outgrowth and patterning of the limbs, IGF-I has been identified in mesoderm regions that undergo programmed cell death, including the interdigital zone in mouse and chicken embryos (52,53,55). Therefore, IGF-1 is implicated in all activities (proliferation, differentiation and apoptosis) essential for proper limb morphogenesis.

Notably, no knock-outs of any IGF-axis member to date have been reported to result in defects in limb initiation, outgrowth or patterning. Thus, although *Igf1*(−/−) and *Igf2*(−/−) mutants exhibited growth impairment, only marginal ossification retardation occurred, and this did not exceed one embryonic day (12,13). However, postnatal comparisons of wild-type and surviving *Igf1*(−/−) mutants revealed the rate of long bone ossification to be greatly reduced in the mutants (14). Ablation of the *Igf1r* gene resulted in a greater delay in the appearance of the ossification centers in facial and cranial bones (lag of ~2 embryonic days), and ossification of the interparietal bone exhibited an even longer delay (~4 days) (13). In support of these findings, the intravascular infusion of recombinant IGF-I in late gestation of fetal sheep resulted in no change in the lengths of the fetus and long bones. However, a rise in skeletal maturation was observed, as assessed by the acceleration of the appearance of epiphysial centers and the increase in cross-sectional areas of the bones (56). Furthermore, overexpression of IGF-I in mice resulted in disproportionate overgrowth of certain organs but no increase in the length of long bones (57). These observations indicate the existence of redundant mechanisms for the developmental processes of limb morphogenesis, including chondro- and osteogenesis, and/or compensatory actions of IGF-axis members. In this regard, Dealy and Kosher (58) observed that insulin mimics the effects of IGF-I in promoting AER induction and limb outgrowth *in vitro*, and Messiano *et al* (59) demonstrated that hypophysectomized lamb fetuses with normal plasma concentrations of IGF-I and IGF-II exhibited delayed osseous maturation, which was restored by thyroxine administration. These observations suggest that IGFs are not the sole trophic factors involved in fetal skeletal development. More recent studies, using novel methods to visualize and quantify differences in the structure and mineral density of fetal bones in *Igf1*(−/−) knock-out mice compared with bones in wild-type mice, report hypomineralization and differences in bone microstructure, possibly representing impaired remodeling activity in the absence of IGF-I (60,61).

## 5. IGF-I genetic disorders in humans

In the last few years, reports of patients with genetic defects in various components of the IGF-axis have broadened knowledge regarding the role of IGFs in intra-uterine growth and development. The first human case concerning a patient with a homozygous partial deletion (exons 4 and 5) of the *Igf1* gene was described by Woods *et al* (62). This mutation was manifested by severe intrauterine growth retardation [birth weight −3.9 standard deviations (SD), birth length −5.4 SD and microcephalia] and dysmorphic features (micrognathia and bilateral clinodactyly), in addition to postnatal growth failure, including delayed bone age and severe osteopenia. Although the growth impairment of the *Igf1* null patient was relatively more severe than that of the *Igf1* knock-out mice (13), the overall phenotypic features were similar.

Since then, a variety of molecular defects in the *Igf1* gene have been reported, including homozygosity for a missense mutation (18), another missense mutation with a milder phenotype (63) and a nucleotide substitution (polymorphism) (64), which was later found to also occur in healthy controls (65). In 2010, van Duyvenvoorde *et al* (66), described a case of heterozygosity for a frameshift mutation characterized by short stature and microcephaly. Later, using molecular methods, the same group concluded that the short stature of the patients cannot be attributed exclusively to the *Igf1* gene defect but to the combination of the *Igf1* gene with other factors, including placental IGF-I insufficiency and other genetic factors (67). In any case, severe pre- and postnatal growth impairment, microcephaly, dysmorphic features, retarded skeletal maturation, deaf-mutism and mental retardation, though variable in density, appear to be common characteristics of patients with *Igf1* gene molecular defects.

In humans, the potential lethal effect from total loss of IGF-1R may explain why only heterozygous mutations in the *Igf1r* gene have been reported to date. Thus, several cases of either heterozygosity for *Igf1r* gene mutations (68–75) or *Igf1r* gene haploinsufficiency (loss of the distal long arm of chromosome 15q26) have been reported (76–79). All patients exhibit a similar phenotype to patients with mutations in the *Igf1* gene. However, compared with patients exhibiting heterozygosity for *Igfr1* mutations (Igfr1 haploinsufficiency), patients with loss of the *Igf1r* gene tend to have more prominent phenotypic abnormalities, with greater dysmorphic features, an increased delay in motor development and impaired psychosocial skills. The extent to which these features reflect the loss of contiguous genes on chromosome 15 is uncertain. The heterogeneity in the clinical phenotypes of patients with molecular defects in the *Igf1* or *Igf1r* genes may suggest variability in the degree of functional loss of the IGF-I/IGF-1R interaction and/or the involvement of other genetic or environmental factors.

## 6. Conclusion

The two IGFs and the main IGF receptor IGF-1R are indisputably important in embryonic and fetal growth and development, as indicated by *in vitro* findings, *in vivo* experiments with knock-out mice and case reports of patients with molecular defects in the IGF-axis members. Although IGF-II is more abundantly expressed in the serum and tissues of the conceptus than IGF-I, IGF-I appears to be more closely associated with fetal growth in the majority of species. IGF-I is generally considered to affect fetal growth in a dose-related manner, independently of GH. However, controversy remains surrounding the data from *in vitro* and *in vivo* observations, and the exact role of IGFs, as pertains to the prenatal development of the skeleton, remains uncertain. Further investigation is required in fetuses with impaired skeletal development, in the context of fetal growth restriction or skeletal dysplasia, in order to elucidate the role of the IGFs in fetal growth and skeletal development.
